# Cactus Thorn-Inspired Janus Nanofiber Membranes as a Water Diode for Light-Enhanced Diabetic Wound Healing

**DOI:** 10.1007/s40820-025-01904-z

**Published:** 2026-01-06

**Authors:** Mei Wen, Nuo Yu, Xiaojing Zhang, Wenjing Zhao, Pu Qiu, Wei Feng, Zhigang Chen, Yu Chen, Meifang Zhu

**Affiliations:** 1 State Key Laboratory of Advanced Fiber Materials, College of Materials Science and Engineering, Donghua University, Shanghai, 201620 People’s Republic of China; 2 Materdicine Lab, School of Life Sciences, Shanghai University, Shanghai, 200444 People’s Republic of China

**Keywords:** Janus membranes, Biomimetic, Diabetic wound, Self-drainage

## Abstract

Diabetic wounds present challenges in clinical management due to persistent inflammation caused by excessive exudate infiltration. Inspired by the gradient wettability of cactus thorn, this study has devised a biomimetic Janus nanofiber membrane as a water diode, which endows with gradient wettability and gradient pore size, offering sustainable unidirectional self-drainage and antibacterial properties for enhanced diabetic wound healing. The Janus membrane is fabricated by depositing a hydrophilic polyacrylonitrile/chlorin e6 layer with smaller pore sizes onto a hydrophobic poly(ε-caprolactone) with larger pore sizes, thereby generating a vertical gradient in both wettability and pore structure. The incorporation of chlorin e6 in the upper layer enables the utilization of external light energy to generate heat for evaporation and produce reactive oxygen species, achieving a high sterilization efficiency of 99%. Meanwhile, the gradient structure of the Janus membrane facilitates continuous antigravity exudate drainage at a rate of 0.95 g cm^−2^ h^−1^. This dual functionality of effective exudate drainage and sterilization significantly reduces inflammatory factors, allows the polarization of macrophages toward the M2 proliferative phenotype, enhances angiogenesis, and accelerates wound healing. Therefore, this study provides a groundbreaking bioinspired strategy for the development of advanced wound dressings tailored for diabetic wound regeneration.

**Supplementary Information:**

The online version contains supplementary material available at 10.1007/s40820-025-01904-z.

## Highlights

- Photonic-powered Janus membrane with dual-gradient architecture for efficient wound exudate drainage and evaporation.
- Photodynamic–photothermal Janus membrane for enhanced bacterial eradication.
- Multifunctional Janus membrane with dual drainage–sterilization functions accelerates diabetic wound healing via macrophage reprogramming and tissue regeneration.

**Supplementary Information:**

The online version contains supplementary material available at 10.1007/s40820-025-01904-z.

## Graphical abstract

**Figure:**
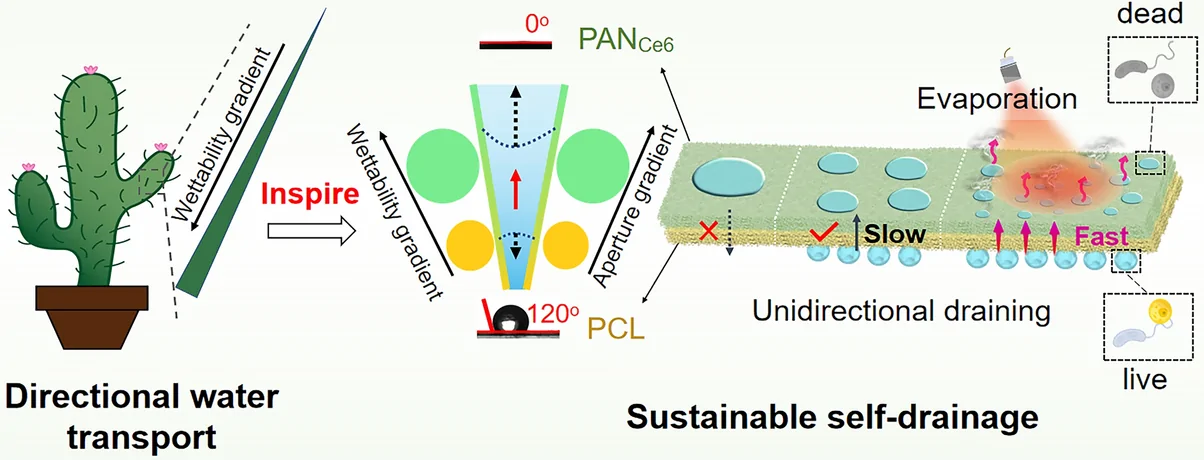

## Introduction

Diabetic foot ulcer (DFU) is a severe complication of diabetes mellitus, posing significant public health concerns worldwide. Epidemiological studies indicate that 19%‒34% of diabetic patients develop DFU, which may lead to limb amputation or mortality in severe cases [1–3]. These chronic wounds are characterized by prolonged inflammatory responses and excessive exudate production under hyperglycemic conditions [4–6]. The continuous exudation of high-sugar interstitial fluid provides a favorable microenvironment for bacterial propagation. In infected wounds with elevated sugar content, oxidative stress gives rise to the generation of excessive reactive oxygen species (ROS), harming islet beta cells and their surrounding tissues [7]. Simultaneously, metabolites like lactic acid and ROS, as well as excessive advanced glycosylation end products (AGEs), trigger a vigorous host immune response, facilitate the release of inflammatory factors, cause oxidative damage, and induce alterations in the extracellular matrix. In addition, this immune response inhibits the proliferation of fibroblasts, endothelial cells, and keratinocytes. Effective exudate drainage during the inflammatory phase of diabetic wound healing is critical for removing pro-inflammatory mediators and cellular debris, thus mitigating excessive inflammation and secondary infections [8–10]. Current clinical strategies for exudate control primarily rely on hydrophilic materials, including medical gauze, polymeric films [11, 12], porous sponges [13–15], and hydrogel-based systems [16, 17]. However, conventional hydrophilic dressings exhibit two major limitations: (1) Their finite absorption capacity leads to rapid saturation, resulting in residual exudate accumulation at the wound site; (2) strong adhesion to nascent granulation tissue often causes mechanical trauma during removal [18]. Consequently, there remains an unmet need for advanced wound dressings that combine high-efficiency exudate clearance with non-adherent properties to support inflammation resolution.

Nature, as the ultimate creator, has inspired numerous innovations through the unique directional water transport capabilities of organisms such as cacti [19], Namib desert beetles [20], and spider silk [21]. These capabilities arise from variations in physical structure and surface energy, driven by asymmetric chemical components or surface morphology, which disrupt contact line symmetry to generate liquid transport forces [19]. Based on these principles, gradient-structured membranes have been developed to enable spontaneous and directional liquid transport. For example, wettability gradients in porous membranes can induce a "smart" directional wicking effect, facilitating unidirectional water transfer [22]. Previous reports have developed hydrophobic-to-hydrophilic gradient fabrics capable of directional water transport from the hydrophobic to the hydrophilic region [22–25]. However, challenges remain due to the trade-off between gradient driving forces and gradient lengths. To enhance directional water transport, two main strategies are employed: pore structure regulation and external energy introduction. Pore structure optimization, particularly through gradient pore sizes between hydrophobic and hydrophilic layers, significantly improves performance due to gradient capillary [26, 27]. Membranes with larger hydrophobic pores and smaller hydrophilic pores achieve higher water collection rates (6.76 ± 0.75 g cm^−2^ h^−1^) compared to the reverse configuration (4.51 ± 0.03 g cm^−2^ h^−1^) [28]. External energy, such as light [29], temperature [30], or magnetic fields [31], further enhances transport efficiency. For instance, photothermal agents improve evaporation efficiency in seawater desalination. Overall, by combining pore structure optimization with external energy, asymmetric wet Janus fabrics offer a promising solution for diabetic ulcer wound regeneration.

**Scheme 1:**
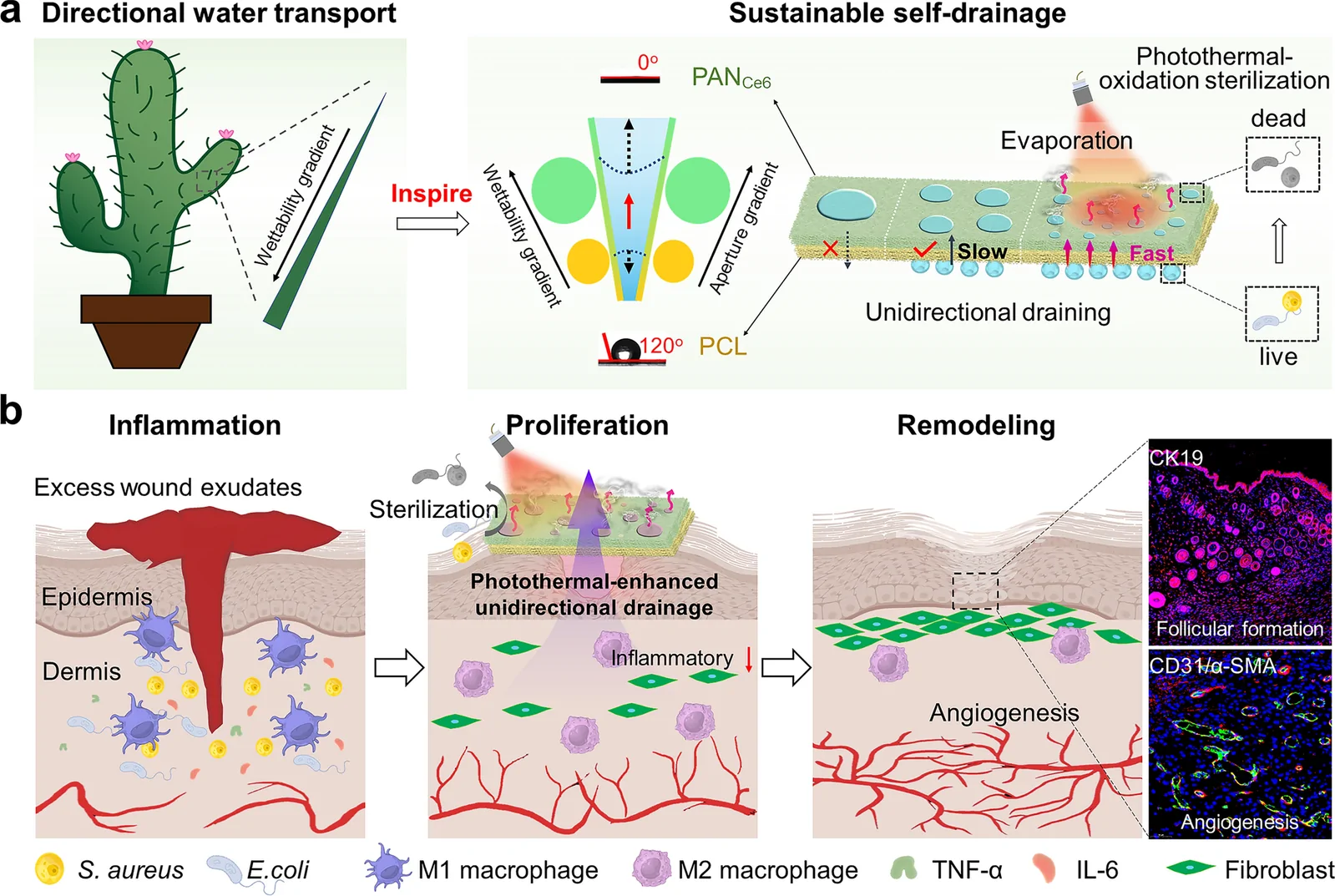
Schematic of **a** fabricating Janus PCL/PAN_x%Ce6_ membrane and **b** wound healing process

Inspired by cactus thorn, herein, we reported biomimetic Janus nanofiber membranes endowed with sustainable self-drainage and antibacterial properties for accelerated healing of diabetic wounds. Specifically, hydrophilic polyacrylonitrile (PAN) fibers containing chlorin e6 (Ce6) were deposited onto hydrophobic poly(ε-caprolactone) (PCL) to manufacture Janus nanofiber membranes (PCL/PAN_*x*%Ce6_) featuring gradient wettability and gradient pore size in the vertical direction (Scheme 1a). Based on the gradient distribution of pore size wettability, water can be spontaneously “pumped” from the bottom hydrophobic layer to the top hydrophilic layer by capillary force and wetting force, resulting in self-drainage. In addition, Ce6, as a photoresponse reagent within the top layer, can generate heat and singlet oxygen (^1^O_2_) for evaporation and photothermal–oxidation sterilization. Evaporation and micro-/nanochannels ensure a continuous water flow with a drainage rate of 0.95 g cm^−2^ h^−1^, conferring the unsaturation state and sustainable pumping as well as a high bacterial eradication rate of 99%. As a result, exudate discharge can promptly remove the high-level inflammatory factors in diabetic wounds in time and ensure the polarization of macrophages toward M2 phenotype, thereby promoting angiogenesis and expediting wound healing (Scheme 1b). This study would offer an approach for fabricating dressings applicable to the regeneration of diabetic wounds.

## Experimental Section

### Preparation of PCL/PAN_***x***%Ce6_

0.5 g of PCL was introduced into a mixture containing 3 mL DMF and 2 mL trichloromethane, obtaining a PCL spinning solution. The spinning fluid of the upper fiber membrane (PAN_*x*%Ce6_) was prepared by adding 0.3 g PAN and different amounts (0, 2.5, 5, and 10 mg) of Ce6 into 3 mL DMF. Subsequently, PCL and PAN_*x*%Ce6_ were electrospinned in turn, resulting in PCL/PAN, PCL/PAN_0.8%Ce6_, PCL/PAN_1.6%Ce6_, and PCL/PAN_3.3%Ce6_ Janus membranes. Electrospinning parameters: The received distance is 15 cm; the spinning voltage is 9 kV, and the pushing speed is 0.04–0.06 mm min^−1^. Finally, the prepared composite fiber membranes were placed in a vacuum oven for 12 h to remove the residual solvent.

### ROS Generation

The production of ROS was detected by the Singlet Oxygen Sensor Green Fluorescent Probe (SOSG). In brief, the PCL/PAN_*x*%Ce6_ (3 mm × 3 mm) were immersed in SOSG solution (2 µM), followed by exposure to 660-nm light (20 mW cm^−2^). Then the fluorescence intensity of the solution at 525 nm was recorded by a fluorescence spectrometer (*E*_*x*_ = 495 nm).

### Photothermal Performance

A 660-nm laser was employed to investigate the photothermal performance of PCL/PAN_*x*%Ce6_. In brief, PCL/PAN_*x*%Ce6_ membranes were cut into small pieces of 1 cm × 1 cm, followed by irradiation with laser at 80 mW cm^−2^. During the irradiation, the temperature change was monitored by an infrared thermal imager. Meanwhile, PCL/PAN_*x*%Ce6_ membranes were soaked in water for 30 s to fully wet the fiber membranes to simulate the wound exudate environment. Then the PCL/PAN_*x*%Ce6_ membranes were exposed to the laser for 180 s to study the photothermal effect under wet conditions.

### Antibacterial Property

The antibacterial properties were studied using models of Escherichia coli (*E. coli*, ATCC 25922) and Staphylococcus aureus (*S. aureus*, ATCC 25923). The antibacterial activity was determined by the plate counting method. Before experiments, the membranes were sterilized with ultraviolet light for 30–60 min. Then the membranes were immersed in 2 mL of bacterial suspension with a concentration of 2 × 10^6^ CFU mL^−1^ and cultured in an incubator at 37 °C. After 30 min of incubation, the membranes were washed with PBS and laid flat on a 12-well plate. The PCL/PAN_1.6%Ce6_ membranes were subjected to four treatments: no light exposure (Control), red light irradiation (5 min, 20 mW cm^−2^), 660-nm laser irradiation (10 min, 80 mW cm^−2^), co-irradiation with red light (5 min, 20 mW cm^−2^), and 660-nm laser (10 min, 80 mW cm^−2^). The bacteria on the membranes were shaken off and diluted 100 times. Then 50 μL of the diluted bacteria were suspended and evenly coated on a fresh AGAR medium (Luria Bertani), followed by culturing in an incubator. Antibacterial efficiency was calculated by the following formula: Bacterial survival rate = *N*_t_/*N*_c_ × 100%, where *N*_t_ and *N*_c_ represent the number of colonies of bacteria in the treatment groups and control group, respectively. The bacterial morphology and activity after treatments were studied by SEM and live/dead staining, respectively. In brief, the PCL/PAN_*x*%Ce6_ with bacteria were washed with PBS and immersed in the 2.5% glutaraldehyde overnight to fix the bacteria. Then these samples were dehydrated with different concentrations of ethanol (20‒100%) and observed by SEM. In addition, the treated bacteria co-stained with PI/Syto 9 to vividly show bacterial activity.

### Inflammation Evaluation In Vivo

The wound tissues were harvested, chopped, digested by trypsin/collagenase II/DNase I, and ground with a nylon filter, yielding a single-cell suspension. After washing with PBS, the cells were stained by fluorescein-labeled antibodies including Anti-CD11b/Anti-CD206 or Anti-CD11b/Anti-CD86, followed by detection with flow cytometry. To assess inflammation levels, the tissue was shredded at 4 °C, frozen by grinding liquid nitrogen, and mashed several times. Then the tissue was added with 500 μL RIPA lysate containing protease/phosphatase inhibitors and incubated in the ice bath for 30 min, fully lysing tissue cells. After ultrasound 10 s, the samples were placed on the ice for another 30 min and centrifugated at 12,000 r min^−1^ for 15 min. The protein expression including interleukin-6 (IL-6) and tumor necrosis factor-α (TNF-α) was determined by western blotting. The β-actin was set as an intrinsic reference protein. Meanwhile, the tissues were also fixed with 4% paraformaldehyde, embedded with paraffin, and cut into slices for histological analysis. The histological analysis included hematoxylin and eosin staining (H&E), Masson's trichrome staining, immunohistochemical staining [myeloperoxidase (MPO) and matrix metalloproteinases (MMP-9)], and immunofluorescence staining (iNOS, CD206, CD11b/c, α-SMA, CD31, and CK19).

### Statistical Analysis

Experiments were carried out at least three times for statistical analysis. The paired Student’s t test was used to estimate statistical significance. In these cases, *p < 0.05, **p < 0.01, ***p < 0.001, were considered statistically significant.

## Results and Discussion

### Preparation and Characterization of PCL/PAN_***x***%Ce6_

**Fig. 1:**
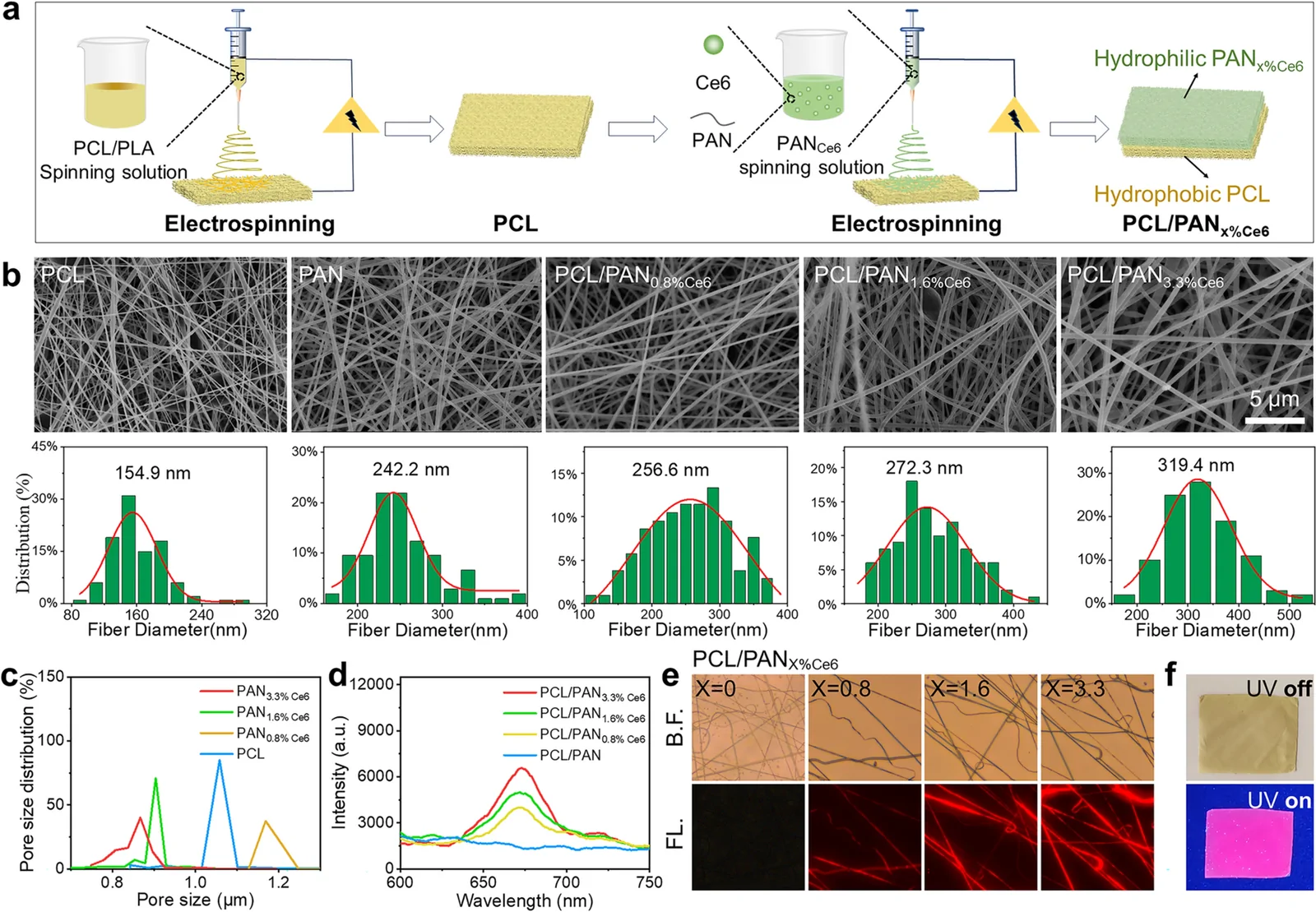
**a** Synthesis process diagram. **b** SEM and fiber diameter distribution of PCL, PAN, PCL/PAN_0.8%Ce6_, PCL/PAN_1.6%Ce6_, and PCL/PAN_3.3%Ce6_. **c** Pore size distribution of PCL and PAN_*x*%Ce6_ (*x* = 0.8, 1.6, and 3.3). **d** Fluorescence spectra of PCL/PAN_*x*%Ce6_ (*x* = 0, 0.8, 1.6, and 3.3). **e** Bright-field (B.F.) and fluorescence (FL.) images of PAN_*x*%Ce6_ (*x* = 0.8, 1.6, and 3.3). **f** Photograph of PCL/PAN_1.6%Ce6_ under 365-nm UV light

The PCL/PAN_*x*%Ce6_ (*x* = 0, 0.8, 1.6, 3.3) nanofibrous membranes were successfully fabricated via electrospinning (Fig. 1a), exhibiting smooth fiber surfaces with uniform diameters of 154.9 nm for PCL and 242.2 nm for PAN (Fig. 1b). Upon Ce6 incorporation, the fiber diameter increased to 319.4 nm due to altered solution viscosity and conductivity, while higher Ce6 concentrations (e.g., 3.3%) induced minor bead formation caused by increased surface tension and Taylor cone instability. The nanofibrous architecture (50–500 nm) outperforms microfibers (> 1 μm) by mimicking native extracellular matrix (ECM) collagen fibrils (50–200 nm) to enhance cell–material interactions, while its nanoscale porosity enables directional fluid transport for the Janus membrane’s "water diode" effect. Furthermore, the membranes possess a well-defined porous structure with pore sizes ranging from 0.8 to 1.2 μm (Fig. 1c), facilitating effective gas exchange for moisture regulation (Fig. S1). Notably, the hydrophilic PAN_*x*%Ce6_ layer (0.9 μm) exhibits smaller pores than the hydrophobic PCL layer (1.1 μm), creating a gradient aperture for wound healing applications. The PCL/PAN_*x*%Ce6_ membrane exhibits good mechanical properties (Fig. S2).

The optical properties of PAN_*x*%Ce6_ fiber films were investigated using UV–Vis–NIR and fluorescence spectrometer. The PCL/PAN membrane without Ce6 exhibits inconspicuous absorption in the visible to the near-infrared region (400–800 nm), whereas PCL/PAN_*x*%Ce6_ (*x* = 0.8, 1.6, 3.3) display enhanced absorption within the range of 400–800 nm, with a dependence on Ce6 content (Fig. S3). After the membrane was soaked in the solution for 6 days, only a small amount of Ce6 is released (Fig. S4). This increased absorption indicates that PCL/PAN_*x*%Ce6_ (*x* = 0.8, 1.6, 3.3) membranes have the potential for a photothermal effect. Under the excitation of 495 nm, PCL/PAN_*x*%Ce6_ (*x* = 0.8, 1.6, 3.3) membranes exhibit a prominent fluorescence peak at 675 nm (Fig. 1d), indicating the presence of Ce6 within the nanofibers [32]. The fluorescence signal from the fibers is visualized using a fluorescence microscope, with red fluorescence uniformly distributed in all Ce6-embedded fibers (Fig. 1e). Additionally, under 365-nm UV light, the membrane displays a pink coloration (Fig. 1f). These results demonstrate the successful preparation of PCL/PAN_*x*%Ce6_ fiber membranes with enhanced light absorption properties.

### Unidirectional Water Transport and Photoresponsive Performance

**Fig. 2:**
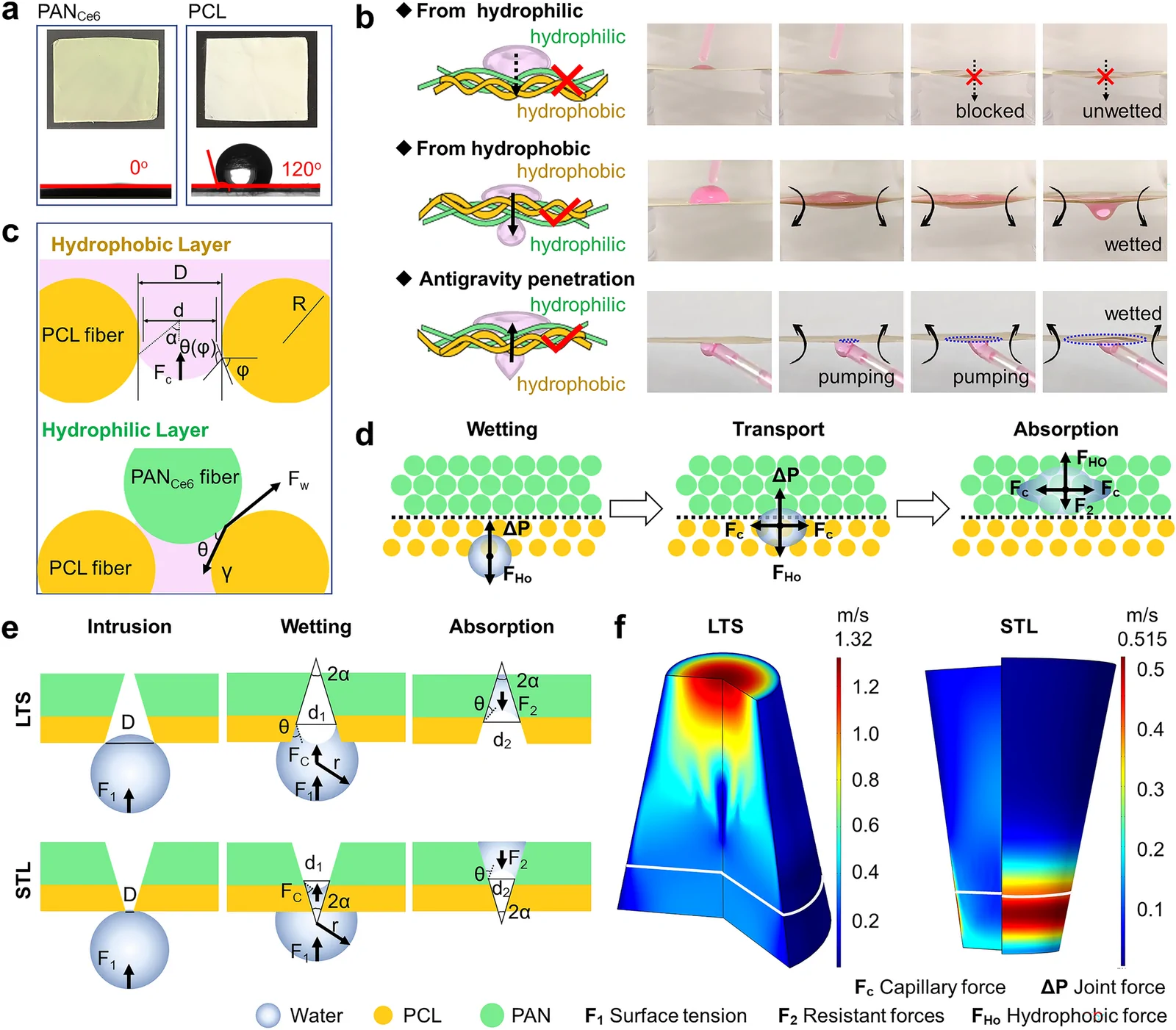
**a** Photographs and the contact angles of the PCL and PAN_Ce6_ layers at 2 s. **b** Schematic and photographs of unidirectional transport processes within PCL/PAN_1.6%Ce6_. **c** Force involved in wetting. **d** Schematic images of the water antigravity penetration. **e** Force analysis of water droplets in large to small (LTS) and small to large (STL) modes. **f** Simulated speed of water transport in the channel

The hydrophilicity of PCL and PAN_Ce6_ layers was investigated by dropping water onto the white PCL or green PAN_Ce6_ surfaces, respectively, followed by measuring their corresponding water contact angles. After 2 s, the PCL layer maintains a contact angle of 110°, reflecting its high hydrophobicity, which suggests its potential to prevent tissue adhesion. Notably, the PAN_Ce6_ layer rapidly reaches 0°, demonstrating its strong inherent hydrophilicity (Fig. 2a). The unidirectional transport processes were investigated by orienting the hydrophilic layer either facing upward or downward and observing the corresponding water-wetting dynamics. When the hydrophilic PAN_Ce6_ layer is positioned upward, it effectively blocks water due to the presence of the hydrophobic PCL, leaving a dry lower layer (Fig. 2b, Video S1). Conversely, when the hydrophilic PAN_Ce6_ is placed as the lower layer, water can be transported from the upper hydrophobic layer to the lower hydrophilic layer (Video S2), demonstrating the unidirectional transport of PCL/PAN_*x*%Ce6_. Furthermore, the antigravity transport performance of PCL/PAN_*x*%Ce6_ was confirmed through experiments in which water was introduced from below. Over time, the water was actively drawn upward by the hydrophilic PAN_Ce6_ layer, resulting in a progressive expansion of the wetting area (Video S3, Fig. S5).

The wetting force was analyzed to elucidate the mechanism of unidirectional transport (Fig. 2c). Upon contact with hydrophobic PCL, the capillary force (*F*_*c*_) is directly proportional to the hydrostatic pressure (HP) affected by surface tension (*θ*(*φ*)), showing a dependence on hydrophobicity [23]. The hydrophobicity-induced *Fc* exhibits resistance to hydrostatic pressure, effectively retaining water and hindering wetting. During spontaneous wetting, water transfers from the hydrophobic to the hydrophilic layer via contact points at the Janus membrane junction. The wetting force (*F*_w_) drives permeation through the hydrophobic layer, initiating hydrophilic layer wetting.

The process of exudate discharge involves antigravity transportation, whose rate depends on the pore size of the membrane. The Janus membranes with pore sizes ranging from large to small (LTS) and small to large (STL) are optimized as quasistatic models for explaining the stress process. When water droplets intrusion the hydrophobic edge of micropores, the micropore size of the contact surface (D) strongly influences the initial intrusion process of the droplet wetting the inner surface of the microchannel (Fig. 2d, e). As micropore size increases, the critical intrusion pressure decreases. Generally, the theoretical intrusion pressure (*P*_0_) of porous materials can be calculated by the following equation:

1 $$P_{0} = \frac{4\gamma }{D}$$

where *γ* is the interfacial tension and *D* is the diameter of the pore in the hydrophobic layer. Thus, the PCL layer with a larger *D* is easier to pass droplets through.

Once penetrating the Janus membrane from the hydrophobic PCL layer, water droplets are subjected to an upward force (Δ*P*) opposing the downward intrusion pressure (*P*₀). When Δ*P* exceeds *P*₀, the droplets advance toward and wet the hydrophilic PAN_Ce6_ layer:

2 $$\Delta P = F_{{\mathrm{C}}} + F_{1}$$

3 $$F_{1} \sim \frac{{D^{2} \pi \gamma_{{{\mathrm{water}}}} }}{2r}$$

where *F*₁ is the droplet surface tension, r is the droplet radius, and $F_{{\mathrm{C}}}$ is the pore capillary force, calculated by:

4 $$F_{{\text{C, LTS}}} = \gamma_{{{\mathrm{water}}}} \pi d_{1} \cdot \cos \left| {\theta - \alpha } \right|$$

5 $$F_{{\text{C, STL}}} = \gamma_{{{\mathrm{water}}}} \pi d_{1} \cdot \cos \left| {\theta + \alpha } \right|$$

where $d_{1}$ is the diameter of the pore in the hydrophilic layer, $\gamma_{{{\mathrm{water}}}}$ is the surface tension of water, θ is Young’s equilibrium contact angle of the hydrophilic coating (0° < θ<90°), and 2α is the conical degree of the conical-shaped microchannel (0° < α<90°). Since $\cos \left| {\theta - \alpha } \right|$> $\cos \left| {\theta + \alpha } \right|$, $F_{{\text{C, LTS}}} > F_{{\text{C, STL}}}$. The larger driving force of the LTS is convenience to wetting.

Subsequently, the peripheral $F_{{\mathrm{C}}}$ drives water spreading across the hydrophilic PAN_Ce6_ layer until complete absorption. Concurrently, *F*_Ho_ diminishes and eventually reverses direction as droplets penetrate the PAN_Ce6_ layer. The resisting forces (*F₂*) during absorption are given by:

6 $$F_{{2, {\mathrm{LTS}}}} = \gamma_{{{\mathrm{water}}}} \pi d_{1} \cdot \cos \left| {\theta + \alpha } \right|$$

7 $$F_{{2, {\mathrm{STL}}}} = \gamma_{{{\mathrm{water}}}} \pi d_{1} \cdot \cos \left| {\theta - \alpha } \right|$$

Since $\cos \left| {\theta - \alpha } \right| > \cos \left| {\theta + \alpha } \right|$, $F_{{2, {\mathrm{LTS}}}} < F_{{2, {\mathrm{STL}}}}$. The smaller resistant forces *F*_*2*_ of LTS would facilitate water absorption process. Therefore, Janus membranes with large to small pore size gradients and wettability gradients from hydrophobic to hydrophilic can act as self-pumps to remove excess biological liquids against gravity.

To further investigate the influence of pore size distribution on the water diffusion rate, COMSOL Multiphysics software was employed to simulate the water transport velocity around the membrane (Fig. 2f). In the simulation, the water transport velocity of the LTS varies from slow to fast from the bottom to the top, with a maximum rate of 1.32 m s^−1^. The LTS mode is conspicuously faster than that of STL mode, where the maximum speed rate of the STL is 0.52 m s^−1^. The rapid diffusion of water in LTS mode is derived from the gradual increment of capillary force.

This "water diode" effect holds significant implications for multiple fiber-related industries, including the medical and textile sectors. In the textile industry, the Janus structure with asymmetric wettability is an ideal candidate for the development of intelligent functional textiles. For instance, it can be used to manufacture sportswear with one-way sweat-wicking capabilities, enabling efficient moisture management during physical activities. Additionally, it is well-suited for creating medical fabrics for wound dressings. In the medical field, wound dressings with the “water diode” effect can greatly enhance the drainage of wound exudate. When choosing a dressing, it is advisable that the aperture of the lower hydrophobic layer is larger than that of the upper hydrophilic layer. Among the available options, the PCL/PAN_*x*%Ce6_ (*x* = 1.6, 3.3) membranes are better equipped to meet these requirements, guaranteeing efficient and controlled fluid management in wound care applications.

**Fig. 3:**
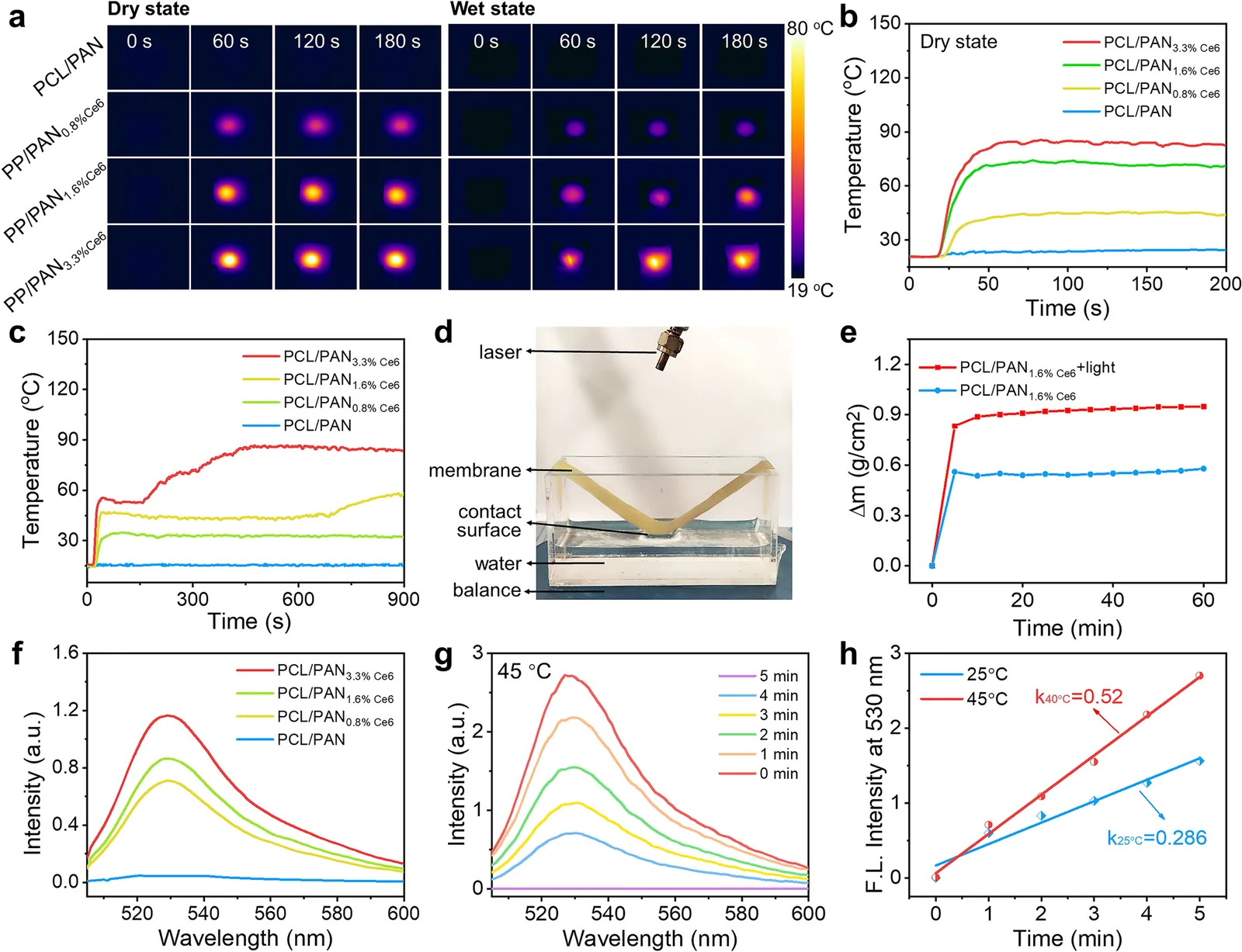
**a** Thermal imaging and **b, c** corresponding temperature curve of PCL/PAN_*x*%Ce6_ (*x* = 0, 0.8, 1.6, 3.3) in the dry or wet state under 660-nm laser irradiation. **d** Picture of a photothermal evaporation device. **e** Evaporation rate of PCL/PAN_1.6%Ce6_. **f** Fluorescence spectra of SOSG after treatment with PCL/PAN_*x*%Ce6_ (*x* = 0, 0.8, 1.6, 3.3) and 660-nm light irradiation. **g** Change in SOSG fluorescence spectra was induced by PCL/PAN_1.6%Ce6_ at 45 °C. **h** Change in SOSG fluorescence intensity at 530 nm was induced by PCL/PAN_1.6%Ce6_ at 25 or 45 °C

The PCL/PAN_*x*%Ce6_ membranes will inherit the photoresponsive ability of embedded Ce6, allowing for light conversion to heat and ROS generation. To confirm the photothermal capability, the temperature change of PCL/PAN_*x*%Ce6_ (*x* = 0, 0.8, 1.6, 3.3) membranes was monitored when exposed to a 660-nm laser (80 mW cm^−2^). The temperature of the dry PCL/PAN membrane remains relatively constant due to the absence of a photothermal agent. In contrast, the temperature of dry PCL/PAN_*x*%Ce6_ (*x* = 0.8, 1.6, 3.3) significantly goes up to 46, 74, and 82 °C, respectively (Fig. 3a, b). The high dry-state temperature may cause partial softening or structural changes in PCL more or less (Fig. S6). Considering the excessive exudation in the wound, PCL/PAN_*x*%Ce6_ (*x* = 0, 0.8, 1.6, 3.3) membranes were immersed in PBS for 30 s to simulate the wound environment. The temperature of wet PCL/PAN_*x*%Ce6_ (*x* = 0.8, 1.6, 3.3) membranes reaches the first platform at 34, 46, and 53 °C, respectively (Fig. 3c). Over time, a second temperature plateau is observed at 58 and 86 °C in the PCL/PAN_1.6%Ce6_ and PCL/PAN_3.3%Ce6_ samples, respectively, which can be attributed to evaporation and subsequent re-increase of temperature. The PCL/PAN_*x*%Ce6_ shows good stability and conversion efficiency (39.4%, Fig. S7). It is evident that PCL/PAN_*x*%Ce6_ has the capability to absorb near-infrared light and convert it into heat for exudate management as well as sterilization purposes.

The photothermal properties of PCL/PAN_*x*%Ce6_ enhance unidirectional drainage speed through thermally accelerated evaporation. When slightly immersed in water and exposed to laser irradiation for 60 min (Fig. 3d), the membrane exhibited a drained water weight of 0.95 g cm⁻^2^, 1.33-fold higher than without irradiation (Δm = 0.71 g cm⁻^2^, Fig. S8). This increased evaporation rate (PCL/PAN_*x*%Ce6_ + light) confirms stable and continuous water evaporation driven by the photothermal effect (Fig. 3e). The PCL/PAN_*x*%Ce6_ membrane thus facilitates sustained unidirectional water expulsion from the inner to the outer layer.

The reactive oxygen species (ROS) generation was investigated at 25 or 45 °C temperatures using an SOSG probe. Upon light stimulation (20 mW cm^−2^), there is enhanced fluorescence intensity in the membranes containing Ce6, indicating the generation of ^1^O_2_ (Fig. 3f). The production of ^1^O_2_ is positively correlated with the concentration of Ce6 doping. Subsequently, the PCL/PAN_1.6%Ce6_ is selected as a model for investigating the impact of temperature on ^1^O_2_ generation. As time prolongs, ^1^O_2_ generation gradually increases with the yield of ^1^O_2_ generation at 45 °C being 1.8 times higher than that at room temperature (25 °C, Figs. 3g, h and S9). Thus, high temperatures can facilitate the ^1^O_2_ production rate.

### Antibacterial Property

**Fig. 4:**
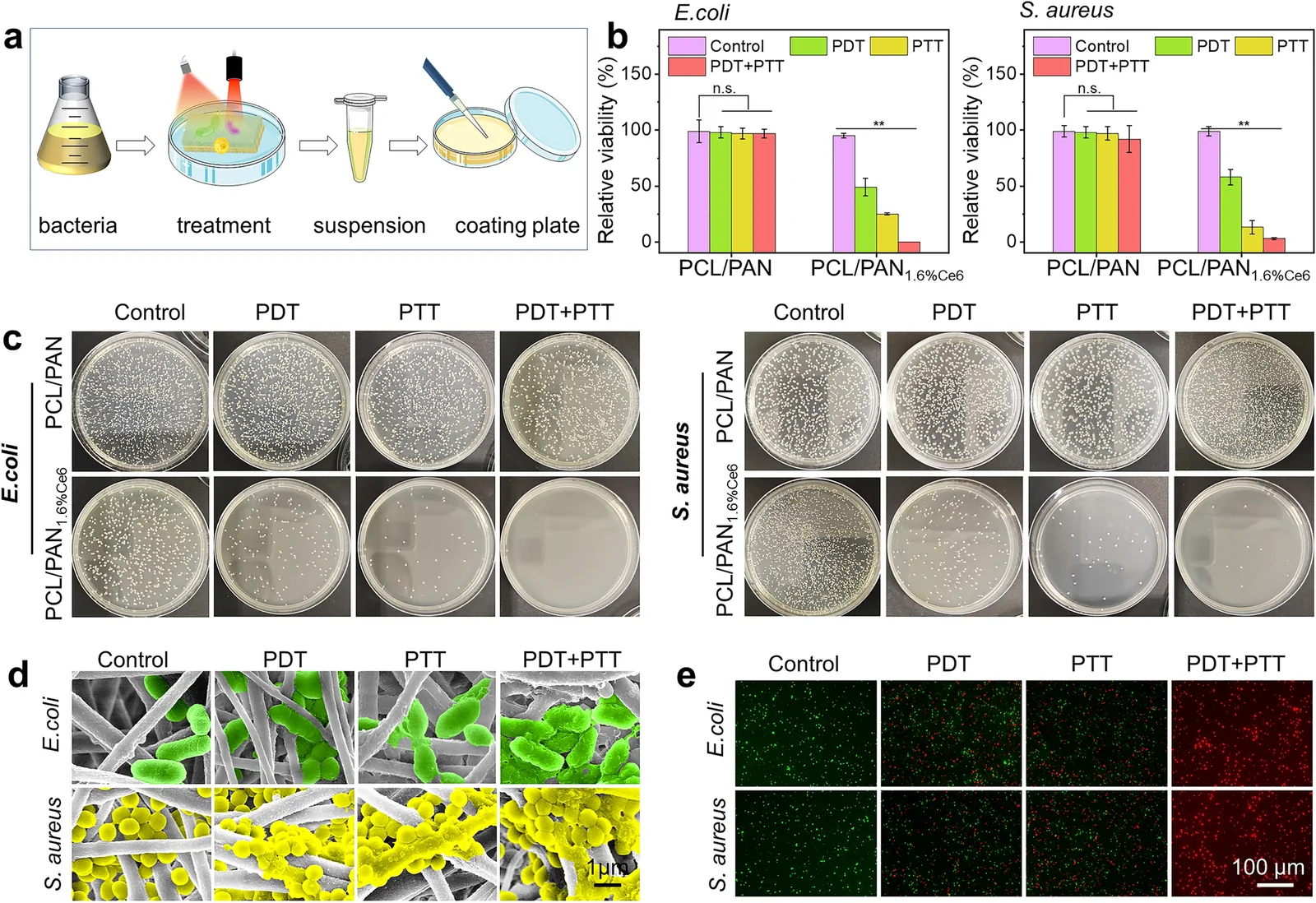
**a** Antibacterial experiment diagram. **b** Survival statistics, **c** photographs, **d** SEM images, and **e** fluorescence images of *E. coli* and *S. aureus* after treatment with PCL/PAN or PCL/PAN_1.6%Ce6_ under light irradiation

The PCL/PAN and PCL/PAN_1.6%Ce6_ were selected as models to validate their antibacterial efficacy. The membranes were immersed in a suspension of Escherichia coli (*E. coli*) or Staphylococcus aureus (*S. aureus*) at a concentration of 2 × 10^6^ CFU mL^−1^, placed in a Petri dish, and exposed to laser or light irradiation. Subsequently, the bacteria detached from the membranes were diluted and plated on LB agar for further enumeration (Fig. 4a). In the absence of light irradiation, both the PCL/PAN and PCL/PAN_1.6%Ce6_ groups demonstrate high bacterial survival rates (> 97%), with intact morphology and smooth surfaces, confirming their lack of inherent antibacterial activity (Figs. 4b–d and S10). In contrast, under light/laser irradiation, the PCL/PAN_1.6%Ce6_ groups exhibit a significant reduction in bacterial colony counts along with noticeable surface shrinkage, demonstrating the effective antibacterial activity mediated by PTT or PDT. After a single round of photodynamic and photothermal treatment, the survival rate of *E. coli* is 48% and 25%, respectively, while that of *S. aureus* is 58% and 14%. When combining PTT and PDT, there is a significant decrease in bacterial colonies, severe damage to bacterial forms, and leakage of bacterial contents. The bactericidal rate for *E. coli* reaches 99.99%, with a similar bactericidal rate of 99% observed for *S. aureus*. The Syto 9/PI staining also confirms the antibacterial effect of PTT and PDT (Fig. 4e). High temperatures and ROS can also cause damage to normal cells (Fig. S11), so attention should be paid to the duration of light exposure. Therefore, the aforementioned result prove that the combined photothermal–photodynamic treatment induces a thermal oxidation effect, leading to localized high temperatures which facilitate the penetration of ROS and subsequent bacterial death, thereby showcasing remarkable bactericidal properties.

### Diabetic Wound Healing and Immunomodulatory

To evaluate the antibacterial efficacy of the membranes, a diabetic wound infection model was established. Diabetic mice with 10-mm surgical wounds were randomly divided into five experimental groups (*n* = 3 per group): I, Control; II, Light; III, bandage; IV, PCL/PAN_1.6%Ce6_; V, PCL/PAN_1.6%Ce6_ + Light. Appropriate dressing regimens were applied to the wounds based on the assigned groups. Groups II and IV were concurrently exposed to the red light (20 mW cm^−2^) and a 660-nm laser (80 mW cm^−2^).

**Fig. 5:**
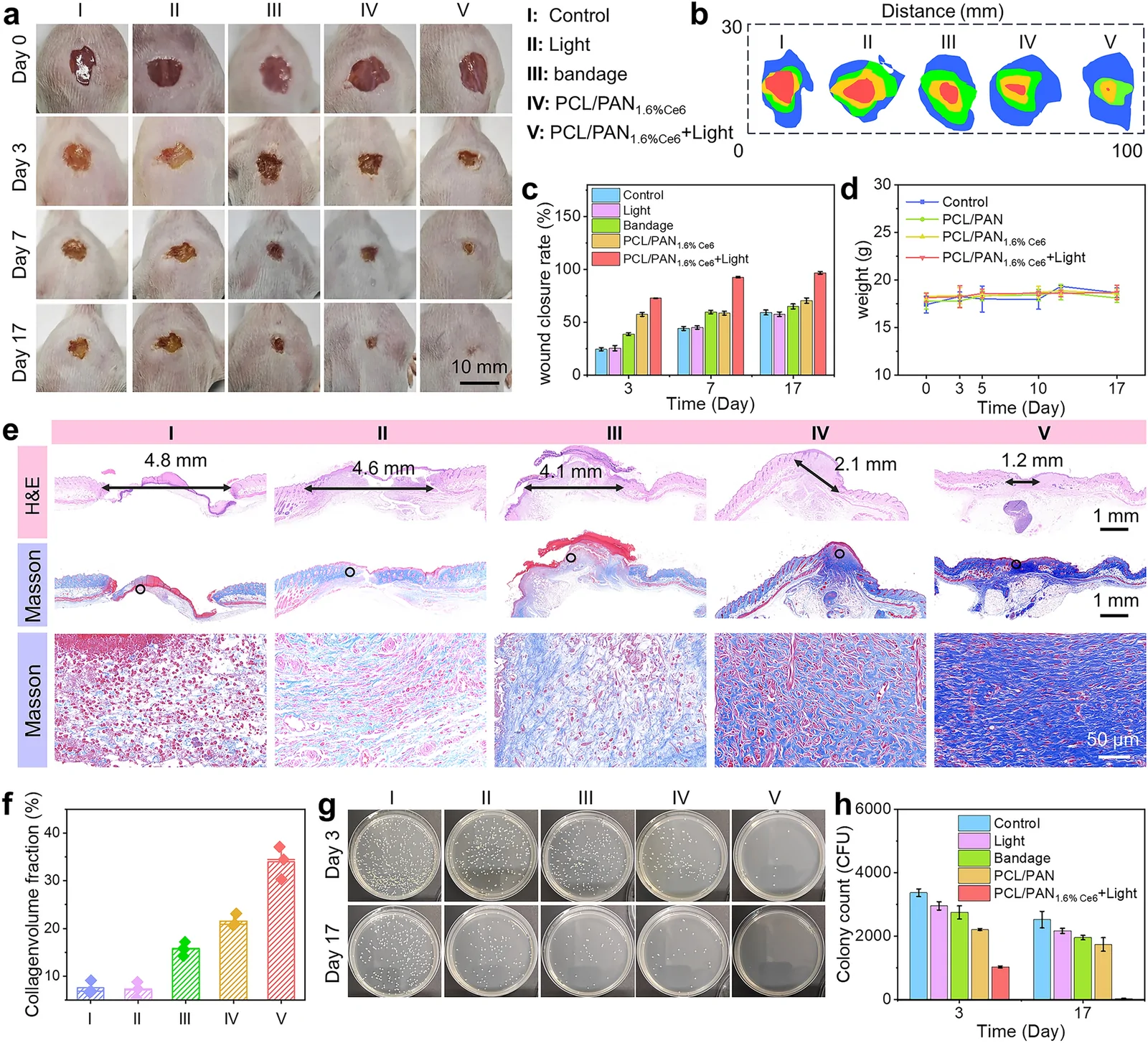
**a** Photographs and **b** area of mice wounds during the healing process. **c** Wound closure rate in each group. **d** Weight of mice. **e** H&E and Masson staining of wound sites. **f** Collagen deposition ratio, **g** photographs of colonies, and **h** statistics at the wound site on days 3 and 17

Wound healing progression was systematically monitored using digital photography on days 0, 3, 7, and 17 for sequential image analysis. Quantitative assessment reveals significantly accelerated wound contraction in the PCL/PAN_1.6%Ce6_ + Light group compared to gauze, light-only, and PCL/PAN_1.6%Ce6_ treatments (Fig. 5a, b). By day 3, the wound healing rates reach 38% (bandage), 57% (PCL/PAN_1.6%Ce6_), and 72% (PCL/PAN_1.6%Ce6_ + Light), all substantially higher than the control group's 24.5% (Fig. 5c). The therapeutic superiority of the combined treatment becomes particularly evident by day 17 due to photothermal effect (Fig. S12), with the PCL/PAN_1.6%Ce6_ + Light group achieving 96.45% wound closure—markedly surpassing the control (59.85%), light-only (58.2%), bandage (65.31%), and PCL/PAN_1.6%Ce6_ (70.92%) groups. These results demonstrate that the synergistic combination of PCL/PAN_1.6%Ce6_ with light irradiation promotes superior wound repair and skin regeneration, likely mediated through enhanced exudate drainage and combined photodynamic/photothermal sterilization effects. In addition, throughout the treatment period, each group's weight remains stable confirming good biocompatibility of photodynamic/photothermal sterilization (Fig. 5d).

The wound healing on day 17th was evaluated using Hematoxylin and Eosin staining (H&E) and Masson staining. H&E results correlate well with the observed healing rates, demonstrating that the PCL/PAN_1.6%Ce6_ + Light group exhibits the shortest wound length (Fig. 5e). Collagen deposition represents a key biomarker of wound healing progression, especially in diabetic wounds where impaired extracellular matrix remodeling typically results in loosely organized, fragmented collagen fibers. While control groups (I-II) showed minimal collagen deposition (~ 7%), treatment with bandage and PCL/PAN_1__.6%Ce6_ membrane alone improves deposition to 14.7% and 21.6%, respectively (Fig. 5f). Remarkably, the PCL/PAN_1.6%Ce6_ + Light combination induces robust collagen deposition (34%), with histomorphometric analysis revealing dense, well-organized wavy collagen fibers. To assess the in vivo anti-infective activity, a sterile cotton swab was utilized to sample the wound on both the 3rd and 17th days. After cultivation in an incubator, it is observed that the PCL/PAN_1.6%Ce6_ + Light group shows a significantly lower bacterial count compared to the control group (Fig. 5g, h). This anti-infective effect is anticipated to facilitate expedited healing of diabetic wounds afflicted with bacterial infection. Thus, PCL/PAN_1.6%Ce6_ membrane with unidirectional water transport and photothermal/photodynamic functions demonstrates promising potential for tissue repair applications.

**Fig. 6:**
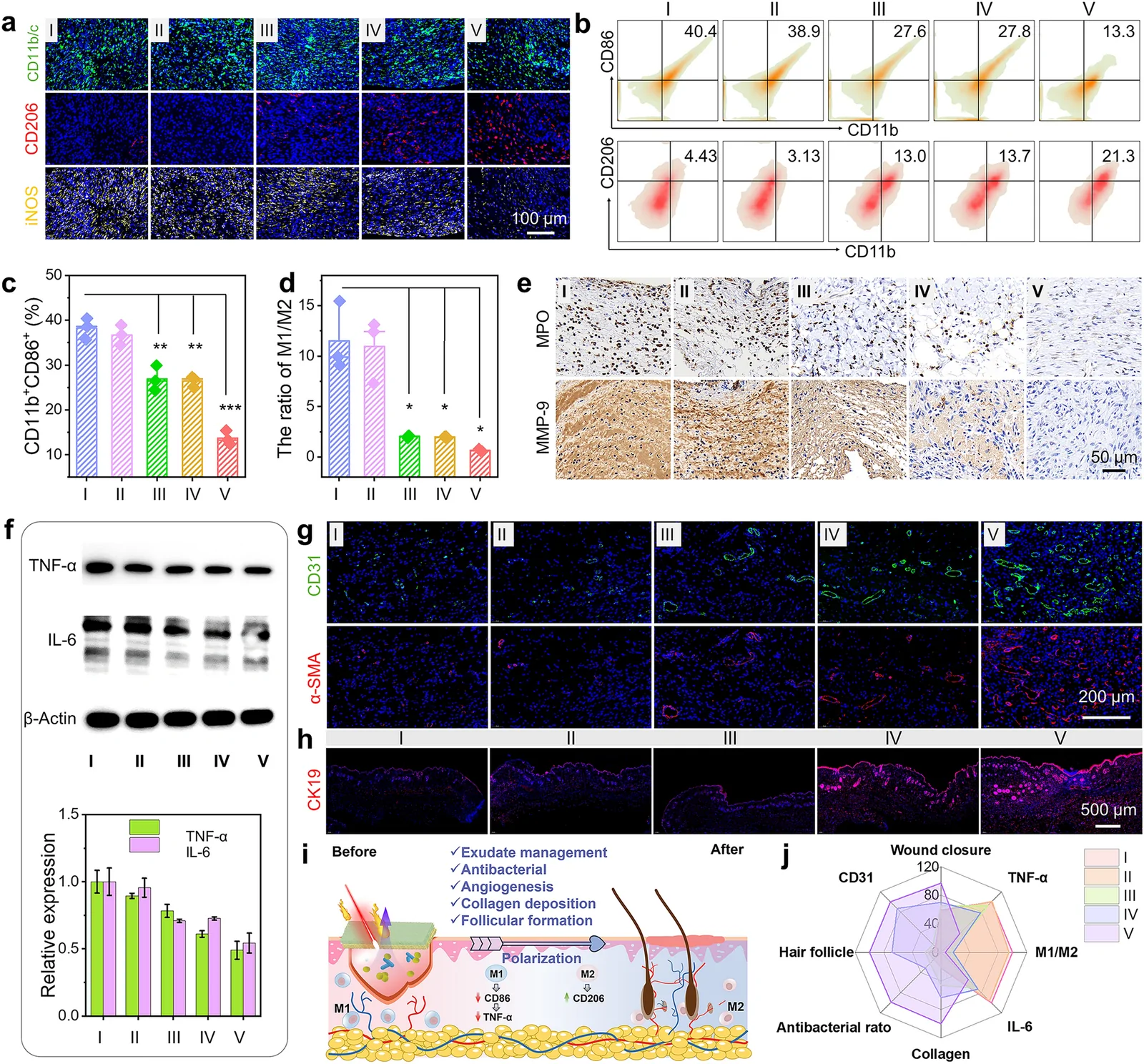
**a** Immunofluorescence staining of CD11b/c, CD206, and iNOS within wounds on day 7. I: Control, II: light, III: bandage, IV: PCL/PAN_1.6%Ce6_, V: PCL/PAN_1.6%Ce6_ + Light. **b** Flow cytometry of macrophage phenotype in the skin tissues. **c** Proportion of M1 phenotype macrophage. **d** The ratio of M1/M2. **e** MPO and MMP-9 immunohistochemical staining images. **f** Western blot and relative expression of TNF-α and IL-6. Immunostaining of **g** CD31/α-SMA and **h** CK19 at wounds on day 17. **i** Operational mechanisms of PCL/PAN_1.6%Ce6_ in diabetic wound healing. **j** A radar chart illustrating key performance indicators such as antibacterial activity, immune modulation, collagen production, vascularization, and tissue regeneration

Persistent inflammation impedes vascularization and delays diabetic wound healing. The balance between M1 (pro-inflammatory) and M2 (pro-reparative) macrophage polarization critically regulates immune responses during wound recovery [33, 34]. Immunostaining on day 7 revealed inflammatory cells (CD11b/c⁺), M1 macrophages (CD11b/c⁺iNOS⁺), and M2 macrophages (CD11b/c⁺CD206⁺). Control groups (I-II) exhibit widespread inflammation, dominated by M1 macrophages (Figs. 6a and S13). Both the bandage and PCL/PAN_1.6%Ce6_ membrane moderately increase M2 macrophage infiltration. Flow cytometry quantified macrophage phenotypes using CD11b/CD86 (M1) and CD11b/CD206 (M2) markers. The percentage of M1 macrophage in control (I), Light (II), bandage (III), PCL/PAN_1.6%Ce6_ (IV), and PCL/PAN_1.6%Ce6_ + Light (V) groups is determined to be 38.2%, 36.7%, 26.9%, 26.4%, and 13.7%, respectively (Figs. 6b, c and S14). The M1/M2 ratio sharply declined from 11.5 (I) to 0.7 (V), demonstrating that PCL/PAN_1.6%Ce6_ + light synergistically promotes M2 polarization (Fig. 6d). The phenotypic transformation of macrophages should be ascribed to the infection clearance and exudate discharge, facilitating tissue repair.

The inflammation severity was further assessed on day 7, including an assessment of the inflammatory indexes and levels of pro-inflammatory cytokines. Myeloperoxidase (MPO) serves as a direct indicator of neutrophil infiltration, while matrix metalloproteinases MMP-9 are secreted as a result of inflammatory cell infiltration [35]. The expression of MPO and MMP-9 was evaluated through immunohistochemical staining. The PCL/PAN_1.6%Ce6_ with light irradiation effectively reduces the expression of MPO and MMP-9 compared to control and traditional bandage groups (Fig. 6e). Meanwhile, the overexpression of IL-6 and TNF-α (key pro-inflammatory mediators) is inhibited (Fig. 6f), indicating that PCL/PAN_1.6%Ce6_ has the potential to mitigate inflammation through effective sterilization and regulation of macrophage heterogeneity.

During the proliferation phase, vascular integrity is crucial for delivering essential nutrients and oxygen to support wound healing, while follicle remodeling and regeneration serve as key indicators of skin repair. On day 17, angiogenesis in diabetic wounds was assessed via immunofluorescence staining. As shown in Fig. 6g, CD31 (green fluorescence, marking neovascularization [36]) and α-smooth muscle actin (α-SMA, red fluorescence, indicating mature vessels) were detectable across all groups (Fig. S15), reflecting ongoing healing. Notably, wounds treated with PCL/PAN_1.6%Ce6_ + Light exhibit more mature vessels, greater vessel length, and larger luminal diameters, suggesting enhanced vascular regeneration. Follicle regeneration was evaluated using CK19 staining, a hair follicle cell marker (Fig. 6h). Immunofluorescence reveals significantly stronger CK19 fluorescence in the PCL/PAN_1.6%Ce6_ + Light group, confirming active follicle formation. Thus, PCL/PAN_1.6%Ce6_, through its dual functions of excess biological fluid removal and photothermal-oxidative sterilization, effectively reduces oxidative stress in diabetic wounds, modulates inflammation, stimulates angiogenesis, and accelerates healing (Fig. 6i, j).

**Fig. 7:**
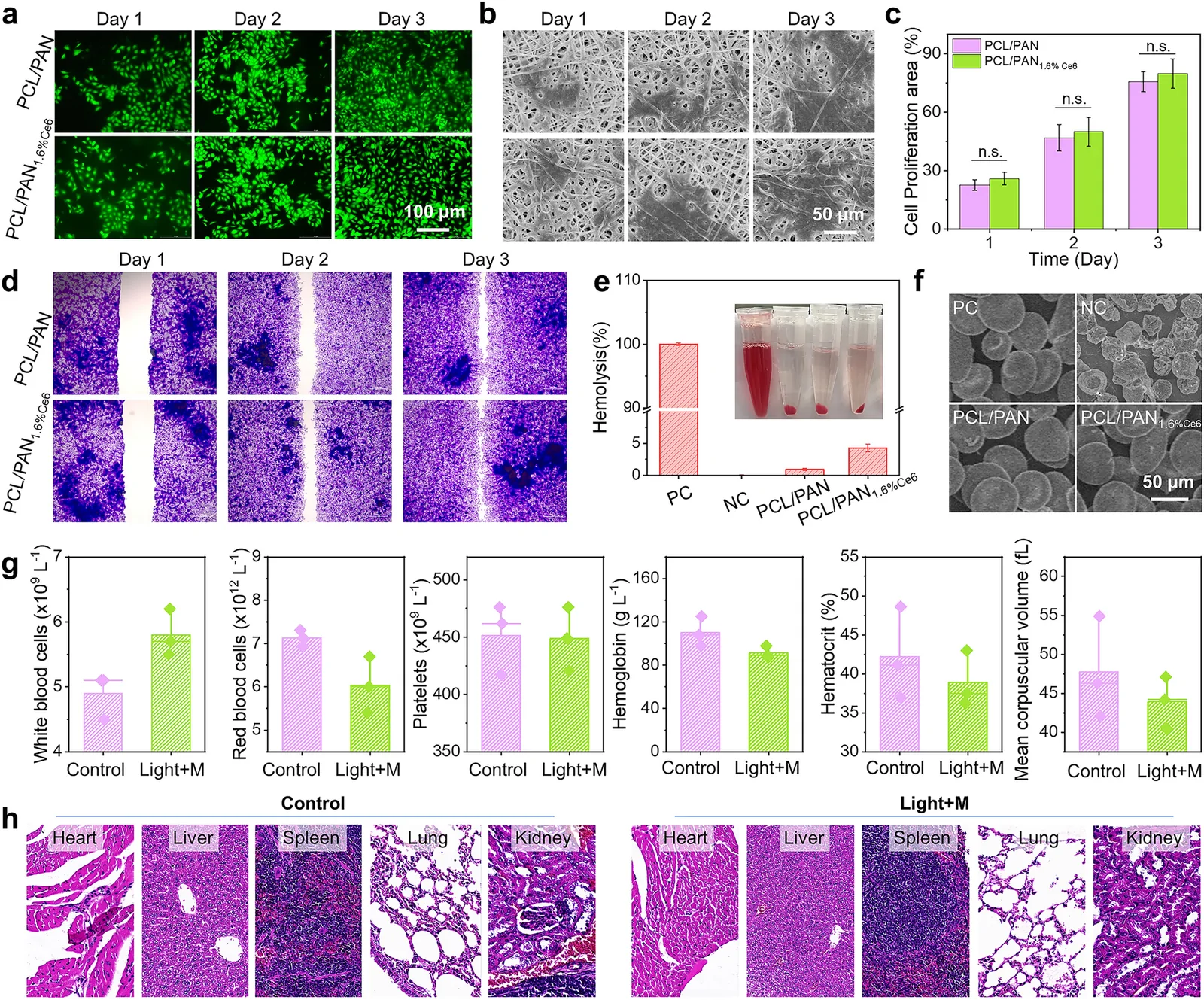
**a** Calcein-AM fluorescent staining images of HUVEC cells. **b** SEM images and **c** proliferation area of HUVEC cells on PCL/PAN or PCL/PAN_1.6%Ce6_. **d** Microphotograph of the HUVEC cell migrations with different treatments. **e** Hemolysis rate of PCL/PAN or PCL/PAN_1.6%Ce6_. **f** SEM images of red blood cells treated with various treatments. **g** White blood cells, red blood cells, platelets, hemoglobin, hematocrit, and mean corpuscular volume of mice from Light + M groups (Light + PCL/PAN_1.6%Ce6_ is abbreviated as Light + M). **h** Main organs sections of mice from control and Light + M groups

The biocompatibility of wound dressings is critical for developing tissue scaffolds and promoting wound healing. The biosafety evaluation of PCL/PAN_1.6%Ce6_ encompasses investigations into cytotoxicity, hemolysis, blood biochemistry, and H&E staining of major organs. The adhesion and proliferation of human umbilical vein endothelial cells (HUVEC) on nanofiber membranes were evaluated through live/dead staining and SEM analysis. HUVEC cells are attached to the nanofiber surface and proliferate rapidly (Fig. 7a–c). In addition, the scratch test proves that the migration ability of HUVEC cells is not significantly affected following treatment with membranes (Fig. 7d). After the incubation with nanofiber membranes, no evidence of hemolysis is observed, with a hemolysis rate lower than 3.5%, and the morphology of red blood cells remains intact (Fig. 7e, f). In the blood assay for the PCL/PAN_1.6%Ce6_ + Light group, no abnormal indices are detected in the blood routine examination (Fig. 7g). Furthermore, there are no discernible histological differences observed in the main organs (Fig. 7h). The biosafety results validate the potential of PCL/PAN_1.6%Ce6_ as a biocompatibility diabetic wound dressing, demonstrating its suitability for further application.

## Conclusions

In summary, we presented a biomimetic Janus nanofiber membrane featuring sustainable self-drainage and antibacterial properties for diabetic wound healing. Inspired by the gradient wettability of cactus thorn, the Janus membrane is meticulously engineered to possess gradient wettability for spontaneous directional water transport. Meanwhile, the gradient pore size and the introduction of external light energy enhance self-drainage ability through capillary force and evaporation. The membrane was fabricated through the deposition of a hydrophilic PAN/Ce6 layer with a smaller pore size on a hydrophobic PCL layer with a larger pore size. Evaporation and micro-/nanochannels facilitate the antigravity pumping of exudates, with a drainage rate of 0.95 g cm^−2^ h^−1^. The Ce6 content enables photodynamic/photothermal sterilization with a 99% eradication rate. The in vivo experiments confirm that PCL/PAN_1.6%Ce6_ membrane can promote macrophage polarization from M1 to the M2 phenotype through infection clearance and exudate discharge, resulting in a 96.5% wound healing rate at day 17. Compared to conventional Janus membranes [22–25], our biomimetic Janus nanofiber membrane integrates gradient wettability and gradient pore size, which provide the driving force for sustainable unidirectional self-drainage. To facilitate clinical translation, future efforts should focus on validating efficacy and safety through systematic clinical trials. This study not only advances the design principles of functional wound dressings but also offers a promising strategy for improving diabetic wound healing.

## Supplementary Information

Below is the link to the electronic supplementary material.

Supplementary file1 (DOCX 4495 kb)

Supplementary file2 (MP4 4109 kb)

Supplementary file3 (MP4 1949 kb)

Supplementary file4 (MP4 3558 kb)
