# Supplementary material for: Cactus Thorn-Inspired Janus Nanofiber Membranes as a Water Diode for Light-Enhanced Diabetic Wound Healing
Source: Nanomicro Lett. 2026 Jan 6;18:101. doi: 10.1007/s40820-025-01904-z (PMC12770207; doi:10.1007/s40820-025-01904-z)
Supplement: Supplementary file 1 — Supplementary file1 (DOCX 4495 kb) [file 40820_2025_1904_MOESM1_ESM.docx]

Supporting Information for

Cactus Thorn-Inspired Janus Nanofiber Membranes as a Water-Diode for Light-Enhanced Diabetic Wound Healing

Mei Wen^1^, Nuo Yu^1^, Xiaojing Zhang^1^, Wenjing Zhao^1^, Pu Qiu^1^, Wei Feng^2^, Zhigang Chen^1,^*, Yu Chen^2,^*, Meifang Zhu^1^

***^1^*** State Key Laboratory of Advanced Fiber Materials, College of Materials Science and Engineering, Donghua University, Shanghai 201620, P. R. China

***^2^*** Materdicine Lab, School of Life Sciences, Shanghai University, Shanghai 200444, P. R. China

*Corresponding authors. E-mail: [zgchen@dhu.edu.cn](mailto:zgchen@dhu.edu.cn) (Zhigang Chen); [chenyuedu@shu.edu.cn](mailto:chenyuedu@shu.edu.cn) (Yu Chen)

**S1 Experiments section**

**S1.1 Materials**

The polyacrylonitrile (PAN, average Mw 149000~151000) and Polycaprolactone (PCL, Mn=50000) were purchased from Aladdin. N, N-Dimethylformamide (DMF, ≥98%), Chlorin e6 (Ce6) were obtained from Shanghai Macklin Biochemical Co., Ltd. Trichloromethane, absolute ethyl alcohol, and dimethyl sulfoxide (DMSO, ≥99.9%) were brought from Sinopharm Chemical Reagent Co., Ltd. Luria-Bertani (LB) broth and LB agar were brought from Shanghai Bowei Technology Co., Ltd. Beyotime Biotechnology provided 3-(4,5-Dimethylthiazol-2-yl)-2,5-diphenyltetrazolium bromide (MTT), Calcein acetoxymethyl ester (Calcein-AM), SYTO 9, propidium iodide (PI), high-glucose Dulbecco’s modified eagle medium (DMEM), and phosphate-buffered saline (PBS).

**S1.2 Physical characterization**

The morphologies of the PCL/PAN_x%Ce6_ samples were characterized by scanning electron microscope (SEM). Pore size distribution of the porous membranes was measured using the Air-liquid test membrane pore size (PSDA-30M) analyzer. The UV-vis-NIR absorption spectra and fluorescence intensity were obtained from a UV-vis-NIR spectrophotometer (integrating sphere attachment) and a fluorescence spectrometer, respectively. The hydrophilicity/hydrophobicity of a single-layer fiber membrane was measured using a contact angle measuring instrument. The unidirectional water transport process of the PCL/PAN_x%Ce6_ fiber membrane was recorded by digital camera.

**S1.3 Force analysis in wetting**

The capillary force (*F_c_*) is calculated by the following equation: F_c_ = s$\frac{4\gamma\sin[\theta\left( \varphi\right)-\varphi]}{d+2R(1-sin\varphi)}$, where *s* denotes the area covered by the fluid, *φ* represents the local geometrical angle, *θ(φ)* is the local contact angle, *R* is the radius of the fibrous membranes, *d* is the interval between two fibrous membranes, and α is the extrusion angle. The wetting force (*F_w_*) can be calculated with the following equation: F_w_ = γn cosθ, where *γ* represents the surface tension of the liquid, *n* is the perimeter of the contact line, and *θ* is the static contact angle of the liquid on the fibers.

**S1.4 Cytotoxicity and proliferation**

The cytotoxicity of PCL/PAN_x%Ce6_ was confirmed using human umbilical vein endothelial cells (HUVECs) as a normal cell model. Cells were cultivated in DMEM containing 10% fetal bovine serum and 1% penicillin-streptomycin at 37 °C and 5% CO_2_ conditions. Cell proliferation on PCL/PAN_x%Ce6_ was assessed through seeding HUVECs on the membranes. After culturing for 24, 48, or 72 h, the membranes were washed by PBS and the cells were stained by Calcein-AM for 30 min. Then cell proliferation was observed by fluorescence microscope and SEM. In addition, the cell viability was determined by MTT assay. Briefly, HUVECs seeded in the 96-well plate were cultured with PCL/PAN_1.6%Ce6_ for 24, 48, or 72 h. After incubation, the membranes were removed from the medium and 100 μL MTT (0.5 mg L^-1^) was introduced into the cells for another 4 h of incubation. Then 100 μL DMSO was added into the mixture with shaking for 10 min. Finally, the cell viability was confirmed according to absorbance at 490 nm. The above experiments were repeated independently three times.

**S1.5 Hemolysis assay**

Blood (10 mg) was obtained from BALB/c mice. The red blood cells (RBCs) were separated by centrifugation at 2000 rpm for 15 min and washed with PBS. Then, RBCs were dispersed in 6 mL of PBS and divided into six equal parts. Each RBCs sample was centrifugated and added to 1 mL of water (positive control), PBS (negative control), PCL/PAN, and PCL/PAN_1.6%Ce6_, respectively. After incubation for 1 h, all the samples were centrifugated at 12,000 rpm for 15 min, and the absorbance of the supernatant at 576 nm was measured using a UV-vis spectrometer. The percentage of hemolysis was calculated by the following equation S2: Hemolysis ratio (%) = (A _sample_ - A _negative_)/(A _positive_ - A _negative_) × 100%. The hemolysis rate was set to 100% in the positive group and 0% in the negative group.

**S1.6 Wound healing**

BALB/c mice (4 weeks old, male) were intraperitoneally injected with streptozotocin (35 mg kg^-1^, 0.1 M citrate buffered saline, pH=4.5). When blood sugar levels exceeded 16.7 mmol L^-1^, the diabetic mice model was considered successful. Subsequently, a circular wound with a diameter of 10 mm was established on the back of each mouse, followed by being infected with 10 μL *S. aureus* (10^8^ CFU mL^-1^), obtaining a diabetic wound infection model. Then the mice with diabetic wounds were randomly divided into five groups: I, Control; II, Light; III, bandage; IV, PCL/PAN_1.6%Ce6_; V, PCL/PAN_1.6%Ce6_+Light. These wounds were treated in different ways, among which groups II, V were co-irradiated with red light (5 min, 20 mW cm^-2^) and 660 nm laser (10 min, 80 mW cm^-2^), group III was covered with bandage, and groups IV-V were covered with PCL/PAN_1.6%Ce6_ membranes. The healing process was monitored by camera and quantified by Image J software. The degree of wound healing was expressed by wound closure rate (CR): CR (%) = A_0_- Ac /A_0_×100%, where A_0_ is the wound closure area on day 0, and A_c_ is the wound closure area on a certain day. In addition, the bacteria were collected from the wound bed on the 3rd and 17th day by sterile cotton swabs. To assess the biosafety, the major organs from control and V groups were harvested for histopathological evaluation through H&E staining. At the same time, venous blood samples from the eye socket were collected and used for blood routine analysis.

**S2 Supplementary Figures**

**
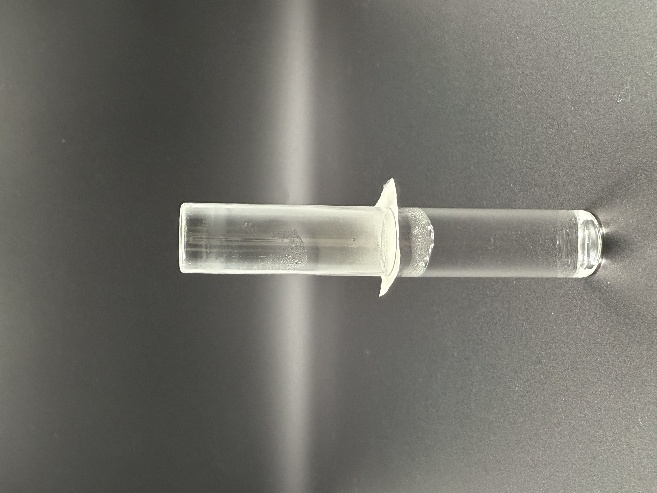
**

**Fig. S1** Evaluation of gas permeability of the PCL/PAN fibrous membranes

**Fig. S2** Force-displacement curves of PAN, PCL and PCL/PAN_1.6%Ce6_ fibrous membranes
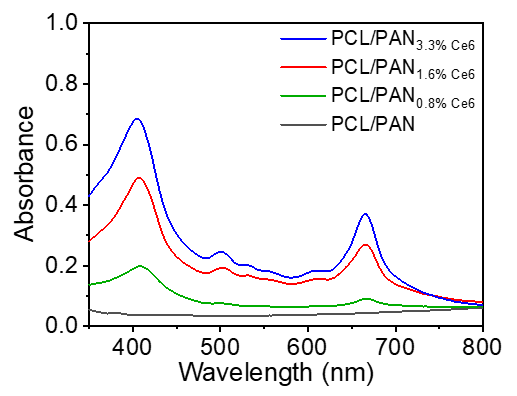


**Fig. S3** Absorbance PCL/PAN_x%Ce6_ (X=0, 0.8, 1.6, and 3.3)


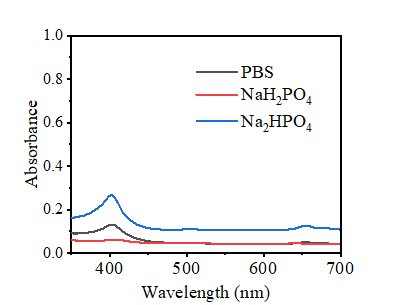


**Fig. S4** The absorbance of the supernatant after the membrane was soaked in the solution for 7 days


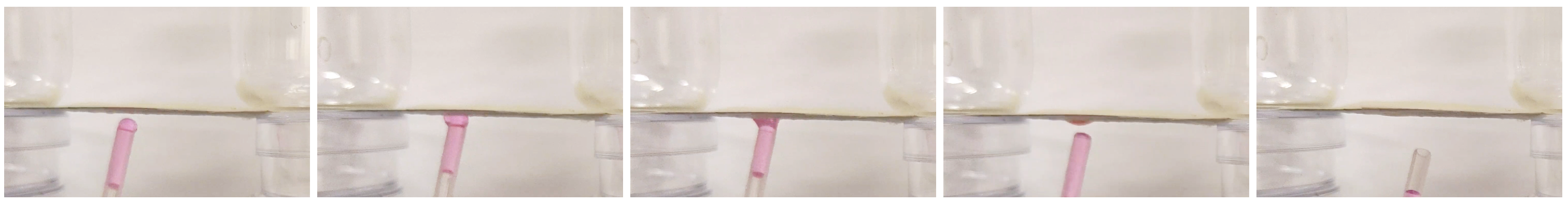


**Fig. S5** Top view of the anti-gravity drainage of PCL/PAN1.6%Ce6 after being exposed to light


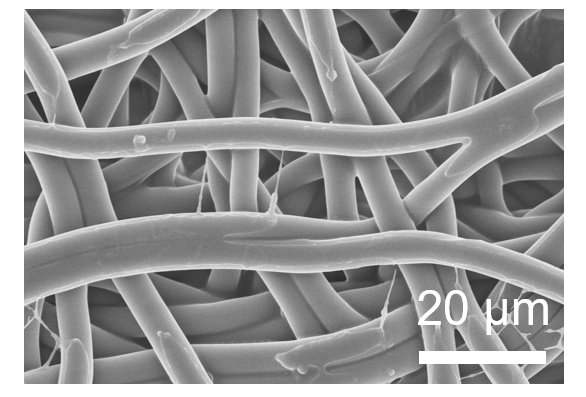


**Fig. S6** The SEM of PCL after irradiation


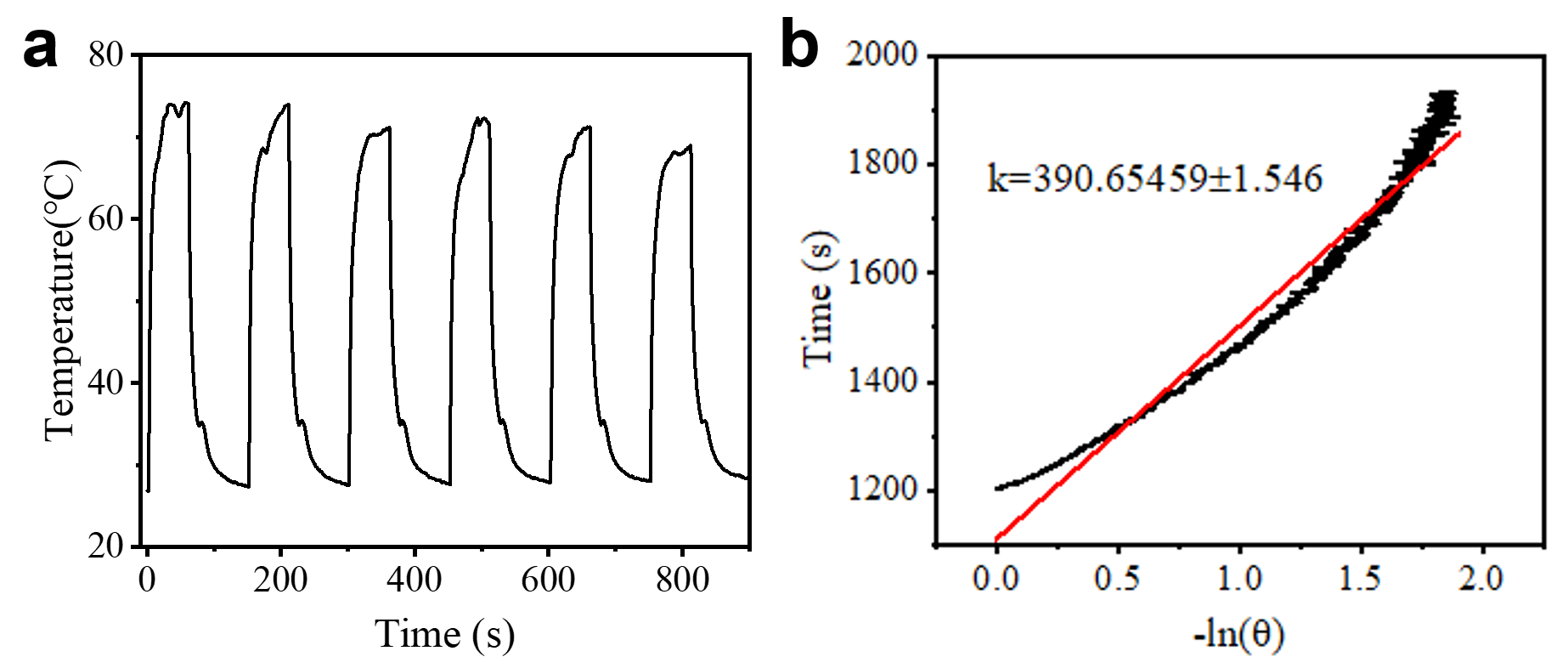


**Fig. S7** (**a**) Photothermal stability during light ON/OFF cycles. (**b**) Time constant s

**Fig. S8** Mass change in the PCL/PAN_1.6%Ce6_ and PCL/PAN_1.6%Ce6_+light groups


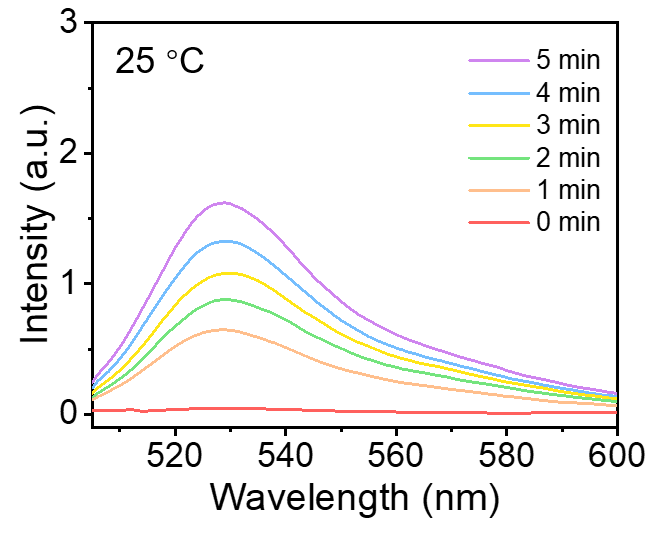


**Fig. S9** The change in SOSG fluorescence spectra was induced by PCL/PAN_1.6%Ce6_ at 25 °C


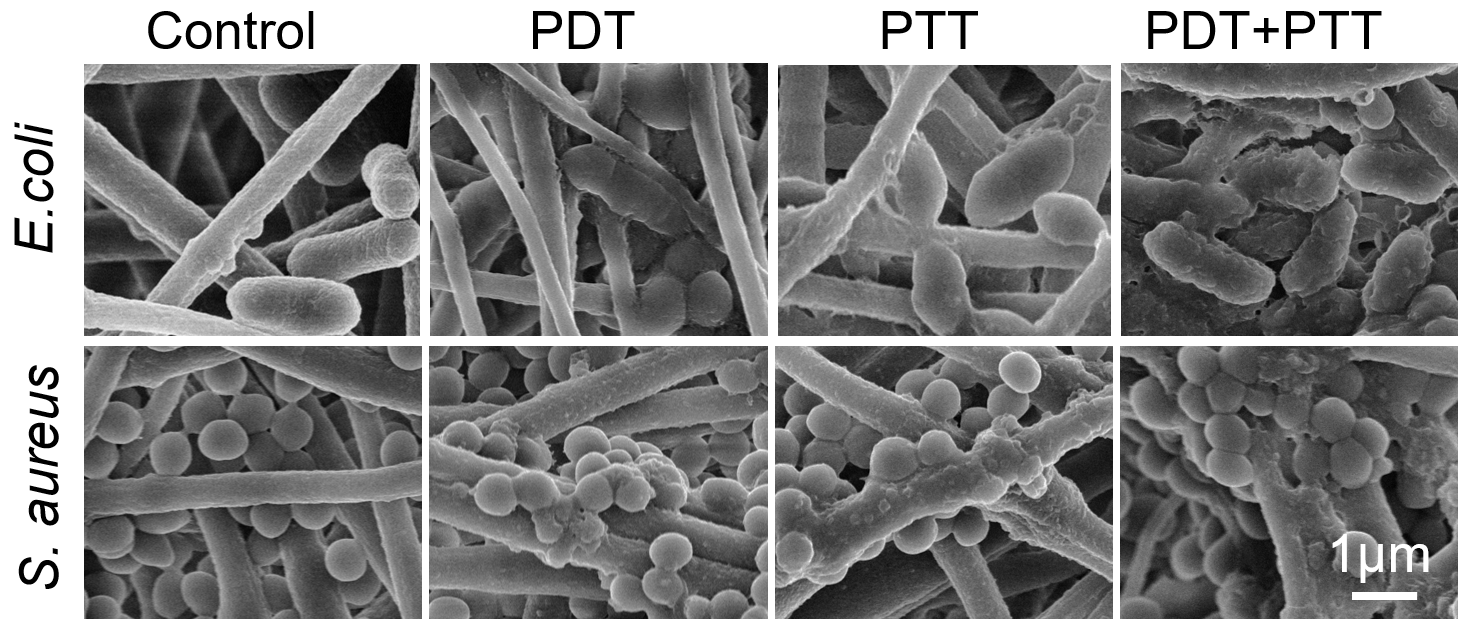


**Fig. S10** SEM images of *E.coli* and *S. aureus* after treatment with PCL/PAN or PCL/PAN_1.6%Ce6_ under light irradiation


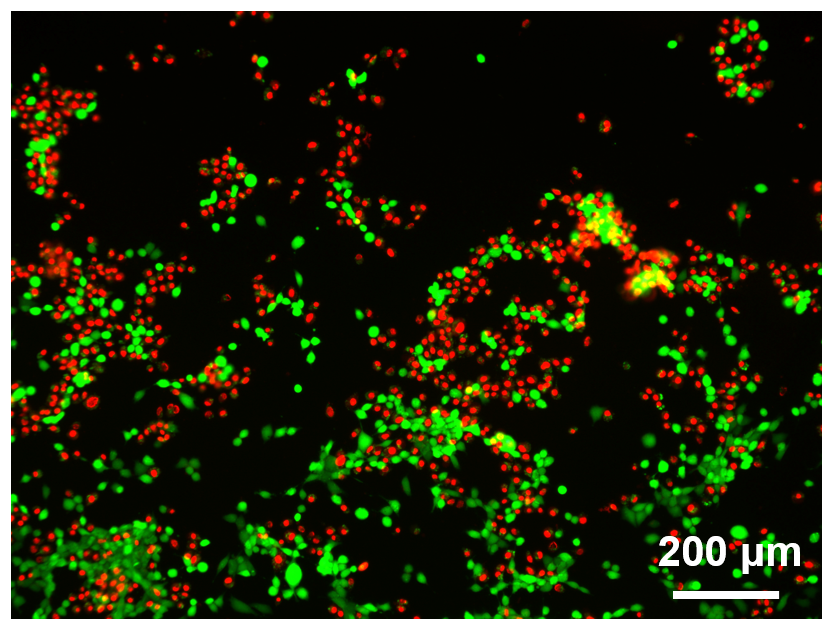


**Fig. S11** Calcein-AM fluorescent staining images of cells


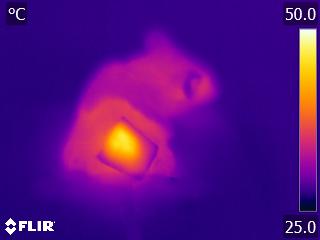


**Fig. S12** Infrared thermal image at the wound sites


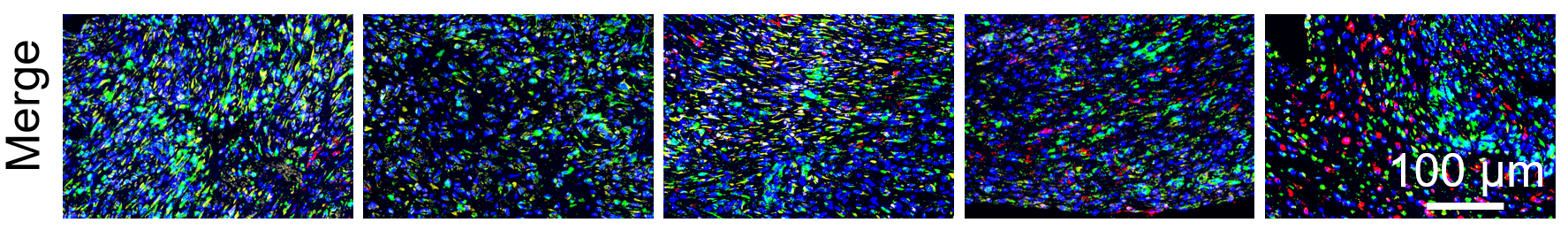


**Fig. S13** Immunofluorescence staining of CD11b/c (green), CD206 (red), and iNOS (yellow) within wounds on day 7


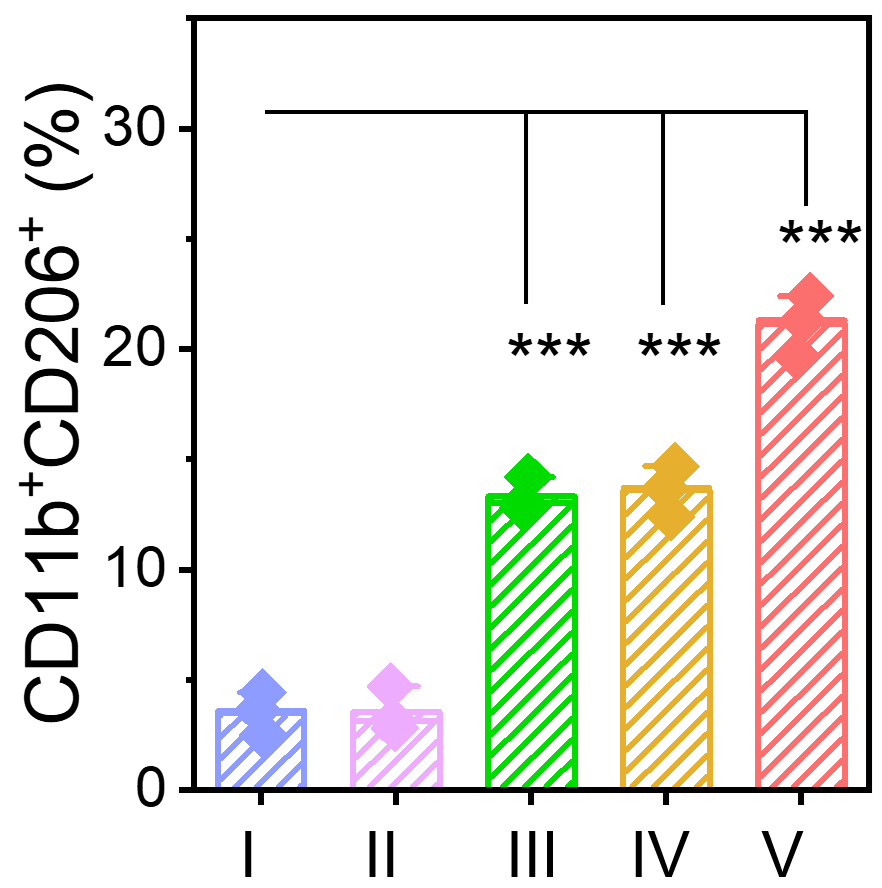


**Fig. S14** The proportion of M2 phenotype macrophage


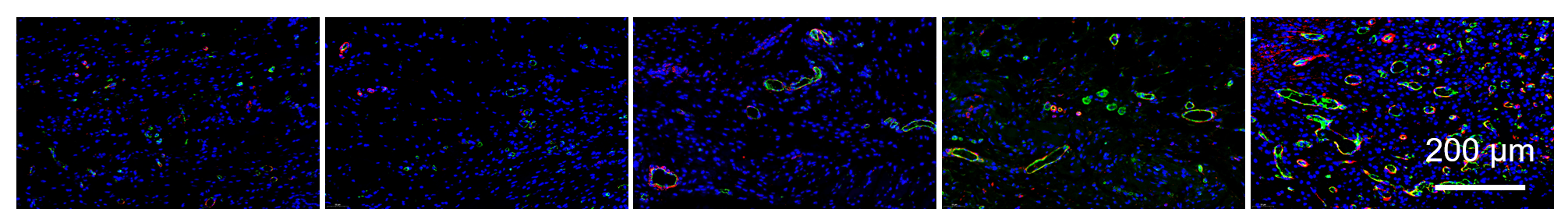


**Fig. S15** Immunofluorescence staining of CD31/α-SMA
